# Factors affecting cognitive remediation outcome in schizophrenia: The role of treatment resistance

**DOI:** 10.1192/j.eurpsy.2021.446

**Published:** 2021-08-13

**Authors:** M. Spangaro, M. Bosia, F. Martini, M. Bechi, M. Buonocore, R. Cavallaro

**Affiliations:** 1 Clinical Neurosciences, IRCCS San Raffaele Scientific Institute, milano, Italy; 2 Faculty Of Medicine, Vita-Salute San Raffaele University, Milan, Italy

**Keywords:** treatment resistance, cognitive remediation, schizophrénia

## Abstract

**Introduction:**

Treatment-resistant schizophrenia (TRS) represents a major clinical issue, characterized by worse psychopathological outcome, a more disrupted neurobiological substrate and higher healthcare costs. Cognitive impairment is a core feature of schizophrenia, strongly associated with patients’ functional outcome. Different studies showed that TRS patients exhibit poorer neurocognitive performance, particularly on verbal domains. To date Cognitive Remediation Therapy (CRT) represents the best available tool for treating cognitive deficits in schizophrenia. However, CRT outcomes are highly heterogeneous and significant treatment predictors are still lacking.

**Objectives:**

To investigate possible differences of CRT outcome among patients with schizophrenia, stratified according to antipsychotic response (TRSs vs. first-line responders - FLRs).

**Methods:**

150 patients with schizophrenia, (95 FLRs, 55 TRSs) were assessed for neurocognition with BACS and WCST at baseline and after CRT. General Linear Models (GLMs) were performed to investigate possible differences between groups on basal cognition and CRT outcome (Cohen’s d Effect Size).

**Results:**

At baseline, GLMs showed significant differences in Verbal Memory (F=4,66; p=0,03) and WCST–executive functions (F=5,59; p=0,02), both worse in TRS group. Effecr Sizes of CRT outcome resulted significantly different in domains of Verbal Memory (F=4,68; p=0,03) and WCST–executive functions (F=4,62; p=0,03), with greater improvements among TRS patients.

**Conclusions:**

This is the first study to indicate treatment-resistance as a possible predictor of CRT outcome in schizophrenia. Moreover, we observed that CRT resulted able to fill the cognitive gap between treatment groups. Thus, these results further highlight the importance of early cognitive interventions in order to reduce the neuropsychological and functional burden associated with the disease, especially for TRS patients.

**Disclosure:**

No significant relationships.

